# Meat and Human Health—Current Knowledge and Research Gaps

**DOI:** 10.3390/foods10071556

**Published:** 2021-07-05

**Authors:** Nina Rica Wium Geiker, Hanne Christine Bertram, Heddie Mejborn, Lars O. Dragsted, Lars Kristensen, Jorge R. Carrascal, Susanne Bügel, Arne Astrup

**Affiliations:** 1 Department of Nutrition, Exercise and Sports, University of Copenhagen, DK-2200 Copenhagen N, Denmark; ldra@nexs.ku.dk (L.O.D.); shb@nexs.ku.dk (S.B.); ara@novo.dk (A.A.); 2 Department of Food Science, Aarhus University, DK-8200 Aarhus N, Denmark; hannec.bertram@food.au.dk; 3 National Food Institute, Division of Food Technology, Technical University of Denmark, DK-2800 Kgs. Lyngby, Denmark; hmej@food.dtu.dk; 4 Danish Meat Research Institute—DMRI Technological Institute, DK-2630 Taastrup, Denmark; lrk@teknologisk.dk; 5 Department of Food Science, University of Copenhagen, DK-1958 Frederiksberg C, Denmark; jorgeruiz@food.ku.dk; 6 IPROCAR, University of Extremadura, E-10004 Caceres, Spain

**Keywords:** fermented meat, processed meat, cancer, cardiovascular disease

## Abstract

Meat is highly nutritious and contributes with several essential nutrients which are difficult to obtain in the right amounts from other food sources. Industrially processed meat contains preservatives including salts, possibly exerting negative effects on health. During maturation, some processed meat products develop a specific microbiota, forming probiotic metabolites with physiological and biological effects yet unidentified, while the concentration of nutrients also increases. Meat is a source of saturated fatty acids, and current WHO nutrition recommendations advise limiting saturated fat to less than ten percent of total energy consumption. Recent meta-analyses of both observational and randomized controlled trials do not support any effect of saturated fat on cardiovascular disease or diabetes. The current evidence regarding the effect of meat consumption on health is potentially confounded, and there is a need for sufficiently powered high-quality trials assessing the health effects of meat consumption. Future studies should include biomarkers of meat intake, identify metabolic pathways and include detailed study of fermented and other processed meats and their potential of increasing nutrient availability and metabolic effects of compounds.

## 1. Introduction

Since ancient times, meat has been a cornerstone of the human diet, and still is in many populations. Even though the amount and source of meat ingested differs between countries and cultures, most Western main meals include a meat-containing dish to which vegetable accompaniments are supplementary. Meat contains several vitamins and minerals, as well as all essential amino acids, making it an excellent protein source [1]. Despite minor differences depending on species and the animal’s diet and age, saturated fatty acids (SFAs) generally constitute almost half the fat in meat, and meat contributes to approximately half of the maximal recommended intake of SFAs [2,3]. The high contribution of SFA has been in the spotlight in recent years, as several large observational studies found positive associations between a high intake of red and processed meat and the risk of cardiovascular diseases, cancer and all-cause mortality, as well as type 2 diabetes [4,5,6]. As a means of reducing the risk of mortality and disease, dietary guidelines have, during the past 30 years, advocated limiting SFA intake to less than 10% of total dietary energy [7,8]. However, SFAs are found in a large selection of foods, varying in their composition with regard to specific SFAs. Furthermore, these foods also differ in structure and content of other nutrients, causing the foods to exert different physiological effects. The current recommendations to reduce SFA intake fail to take into account the different effects of SFAs from different sources [9,10,11].

Risk-of-bias and heterogeneity analyses indicate that the observed link between red and processed meat and an increased risk of disease seen in meta-analyses of observational studies may be due to confounders [12,13,14,15,16]. This highlights, that extrapolation from observational studies should be conducted with caution when evaluating the health effect of meat across populations with major differences in food culture. There is emerging evidence that the specific nutrients in meat may not cause an effect per se, but that the overall composition of the diet and the matrix from the meals are likely to modulate or even cause the observed adverse effects. Several factors, including fiber [17], calcium [18], and cooking practices [19], are likely to be strong effect modulators when investigating meat and disease, and study quality and the inclusion of factors related to the different food cultures surrounding meat intake are likely to play a role as well [15]. This may also include probiotic metabolites from the fermentation of meat, potentially exerting physiological and biological effects, yet unidentified.

The aim of the present paper is to present and discuss the current knowledge and to identify research gaps when assessing the health effects of meat in the human diet.

## 2. Meat as a Source of Nutrients

### 2.1. Amino Acids

With meat being compositionally equivalent to human skeletal muscle, it supplies us with amino acids, having an optimal composition for the support of protein synthesis for building and maintaining muscle. Support and maintenance of skeletal muscle mass is of utmost importance for maintaining both physical function and metabolic health. In alignment with this, meat constitutes an important part of the diet for the elderly to prevent age-related declines in muscle strength and frailty (sarcopenia). Thus, an inverse association between the intake of animal protein and the incidence of frailty was observed in a cohort of 1822 older subjects followed for 2–4 years [20]. In younger and physically active subjects, meat protein intake was recently documented to have direct beneficial effects on body composition and muscle strength [21]. While protein quality is commonly evaluated based on the content of essential amino acids, the bioavailability and bio-accessibility of amino acids are also decisive for the nutritional value of proteins. Hodgkinson and colleagues found that raw meat has a Digestible Indispensable Amino Acid Score (DIAAS) value of 97, while boiled and pan-roasted meat have similar DIAAS values of 99 and 98, respectively. In roasted and grilled meat, the DIAAS is reduced to 91 and 80, respectively [22]. A sophisticated isotope-labelling study revealed higher bioavailability of amino acids from well-cooked meat (cooking at 90 °C for 30 min) than raw meat (cooking at 55 °C for 5 min) when ingested by elderly people [23], illuminating the fact that cooking of meat enables strategic modulation of bioavailability.

While meat is a pivotal source of essential amino acids, it also supplies amino acids, amino-acid-derived metabolites and peptides that have important bioactive properties. Thus, taurine, creatine, hydroxyproline, carnosine, and anserine, which are all mainly obtained from meat, have been proposed to exert important physiological functions [24]. Amino acids are fermented by the microbiota into metabolites with potentially positive as well as negative impact on health; this fermentation takes place especially when other substrates are unavailable. The composition of diet and meals are therefore important determinants of the gut environment. Diets low in dietary fiber, dairy and other potentially protective factors but high in protein may result in a pro-inflammatory response locally as well as systemically, leading to higher risk of disease. In an intervention study comparing Mediterranean diets with habitual diets high in meat and low in dietary fiber, the stool, urine and blood metabolite profiles were consistent with a decrease in toxic amino acid metabolites when a varied diet with dietary fiber was introduced [25].

### 2.2. Vitamins and Minerals

In addition to proteins, meat also supplies us with minerals and vitamins, e.g., the average daily intake among British adults of 189 g contributes with approximately 19, 52, 28 and 38% of iron, zinc, selenium and phosphorus, respectively, according to the reference values of heterogeneous groups [2,3,26]. Zinc is difficult to consume in adequate amounts in diets low in animal-based foods. Even though iron is abundant in a variety of foods, its bioavailability is highest when the source is meat. In meat, iron is complexed and present as heme-iron, which has a considerably higher bioavailability than non-heme-iron. Thus, in the small intestine, approximately 23% of heme-iron is absorbed, whereas this is the case for only 2–8% of non-heme iron [27], and red meat therefore remains the best dietary source of iron [28]. In addition to the higher availability of heme-iron, meat also contains other, yet unidentified, factors increasing iron absorption from other foods (also known as the ‘meat factor’) [29,30]. In relation to vitamins, meat is an important source of complex B vitamins. In fact, meat, fish and other animal-derived foods (such as dairy) are the only unfermented foods that naturally provide vitamin B_12_ [3], and meat and meat products contribute with approximately 30% of the total UK dietary intake of vitamin B_12_ [3]. Collectively, this highlights the need for contemplating the profound effects that replacing a balanced omnivore diet with a vegan diet may have on mineral and vitamin status.

### 2.3. Fatty Acids

Generally, as fat in red meat consists of approximately 40% SFAs, 50% monounsaturated fatty acids, 5% trans fatty acids and 4% polyunsaturated fatty acids [26], meat is considered a major source of saturated fat. Previous observational studies have linked saturated fat with an increased risk of cardiovascular diseases and diabetes; however, studies that are more recent indicate that this was likely confounded by industrial trans-fats in margarines. Attempts to reduce SFA in meat have resulted in several successful approaches to modulate the fatty acid composition of pork and beef through strategic feeding strategies [31].

In contrast to monogastric animals (e.g., pigs), the fatty acid composition in meat from ruminants (e.g., cattle) reflects the composition of the diet to a lesser extent due fermentation and biohydrogenation in the rumen. Although a more unsaturated fatty acid profile can be obtained in pork and beef through feeding strategies, increasing the proportion of unsaturated fat often has deteriorating effects on meat quality, as it is found to be more prone to oxidation and has a less firm structure [31], resulting in meat products that are perceived as unacceptable by consumers [32]. Nevertheless, when addressing fat in meat, an often overlooked fact is that meat originating from ruminants also contains conjugated linoleic acid and unique rumen-derived fatty acids such as branched-chain, vaccenic and rumenic acids, which exert physiological activities and thus have been associated with several positive health effects [33]. Early studies indicated beneficial effects in animal studies. However, these ruminant fatty acids are trans-fats that could potentially cause adverse effects as well; still, a number of Cochrane-based meta-analyses indicate an overall neutral effect of ruminant fats on health in human intervention studies [34,35,36,37].

### 2.4. The Nutrient Contribution from Meat

In the Danish National Survey on Diet and Physical Activity 2001–2013, it was shown that meat and meat products (without poultry and fish) contribute significantly to the average Dane’s intake (as % of total intake) of protein (27%), fat (21%), saturated fatty acids (20%), mono-unsaturated fatty acids (26%), vitamin A (40%), vitamin D (16%), thiamine (33%), riboflavin (17%), niacin (27%), vitamin B6 (21%), vitamin B12 (35%), phosphorus (15%), iron (20%), zinc (33%) and selenium (25%) [38]. The contribution from meat to the dietary nutrient intake is higher in men than in women [39,40]. Thus, meat is an important contributor of several nutrients in a general Danish diet, and if the dietary meat content is reduced, it is important to substitute the meat with various foods that can supply the nutrients usually originating from meat. For example, in a plant-based diet with a low meat content, focus should be on replacing the meat with foods that in particular supply high-quality protein, riboflavin, vitamin B_12_ and vitamin D, iron, zinc and selenium [41].

## 3. What Is Fresh and Processed Meat?

Despite clear definitions within the European Union Law [42,43], the definition of processed meat is inconsistent and varies internationally and between studies, which makes interpretation and comparison of results difficult. Most cohort studies agree to define processed meat as meat that is salted, cured, smoked or dried. The definition of red meat, however, in some studies includes processed meat or some types of processed meat, e.g., bacon; this makes it difficult to identify if it is meat per se or the processing that exerts the observed health effects. Processed meat is often associated with industrially produced products that are cured and/or smoked. In private households and in the catering industry, frying and grilling are normal processing steps in producing the final ready to eat product. Even though fried meat is not comparable to industrially processed meat, frying can contribute to the content of carcinogenic compounds in meat.

### 3.1. Industrial Processing of Meat

The industrial production of processed meat products originates from three fundamental technologies for preservation of meat that were discovered in ancient time, i.e., drying, curing, and smoking [44,45]. Evidence indicates that the practice of hanging meat free for ventilation and thereby removing moisture from the surface decreases the water activity and thereby prevents spoilage bacteria from growing on the meat. Curing by rubbing meat with salt dates back more than 5000 years and due to nitrate-containing impurities in the salt, the shelf life of the meat did not only increase because of salt but also through the presence of nitrite generated from the reduction of nitrate. Salt and nitrite diffuse into the interior of the meat and prolong shelf life by lowering water activity and by means of a direct antimicrobial effect of nitrite. By using a wooden fire to dry meat, it might have been discovered that smoking results in an alternative flavor in addition to a longer shelf life. Smoke contains numerous components that inhibit bacterial growth and prevent lipid oxidation, which explains the positive effect on shelf life. These three fundamental preservation technologies (drying, curing, and smoking) combined with heat treatment have, over time, evolved into the different processes that are used today in the meat industry to produce and increase durability in a vast variety of meat products. A newer methodology of meat preservation is by the addition of antioxidants such as ascorbic acid and its salts. The legislation regarding this method is, however, to a higher degree defined by limiting the water activity rather than a health effect [43].

**Figure 1:**
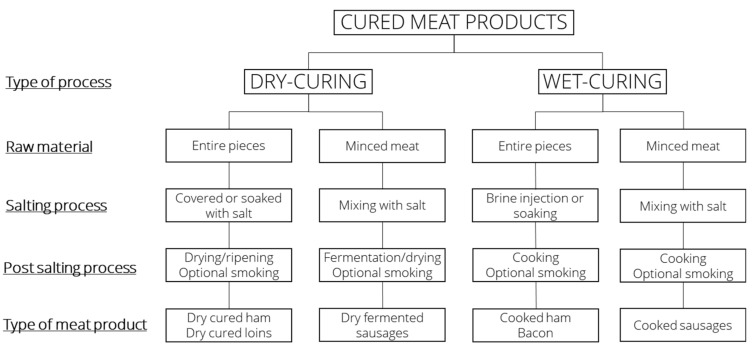
Classification of cured meat products. Adapted from Flores and Toldrá, 1993 [46] and Toldrá, 2017 [47].

Nearly all processed meat products are cured, meaning that salt is added and, in most cases, nitrite or nitrate. Basically, cured meat products can be divided into two main groups based on their respective processes [46]: dry-curing or wet-curing, as illustrated in Figure 1.

### 3.2. Dry Curing

Dry curing involves the use of salt typically in combination with nitrite and/or nitrate, which is rubbed on the surface of entire pieces of meat. The salting process is followed by a drying and ripening period, which runs for several month to years before the product is ready for consumption. Typical products are the Italian Parma and the Spanish Iberico hams. To produce fermented sausages, salt is mixed with minced meat followed by a drying and fermentation period. Spices and bacterial starter cultures are also added to these products to aid in the fermentation process, and especially in the northern part of Europe, the products are also smoked. In the United States, the drying process of fermented sausages is often limited, and the products are cooked [47].

### 3.3. Wet Curing

Wet curing of entire pieces, e.g., cooked ham/loin and bacon, typically involves the use of needle injection of brines containing salt, nitrite, ascorbate and often also phosphates. The diffusion of salt is accelerated by physical treatment in a process known as tumbling, optionally smoked and the product is cooked. An exception is bacon, which is dried for a short time, mildly heat treated, and/or smoked [47]. So-called enhanced meat, where the meat receives added water containing salt and is sold as ‘fresh’ meat, is also within this category, although the consumer performs the cooking process. To produce wet-cured products of minced meat, e.g., cooked sausages, salt and nitrite is mixed with minced meat, added water, spices and ascorbate, filled in casings, and cooked (optionally smoked). Typical products are wieners, mortadella, and frankfurters.

## 4. Maturation and Fermentation

A significant amount of meat is consumed worldwide after a maturation process, including dry-ageing, dry-curing and dry-fermenting. Whereas these processes were historically designed to preserve meat, nowadays they aim for producing a variety of highly delicious products. The ripening process leads to the hydrolysis of certain components such as proteins and lipids, and the formation and release of low molecular weight compounds, both volatile and non-volatile, which give these products an intense and characteristic flavor [48]. There is a huge diversity of meat products of these types all around the world, but they share some common points that are of interest for their potential health outcomes: (1) they include a considerable strong dehydration, up to more than 50% weight loss for some products; (2) they imply significant chemical and biochemical transformation of meat components, including protein and lipid hydrolysis, protein and lipid oxidation and Maillard type reactions as most relevant ones; (3) the process for most of them includes the addition of sodium chloride and nitrates and/or nitrites; (4) most of them undergo extensive microbial transformations by different bacteria, mold and yeast species; this microbiota contributes to acidification, formation of nitrosomyoglobin, proteolysis, lipolysis and flavor formation, to mention their main roles.

While all these changes are directed to obtain a shelf-stable flavorful product with a particular chewy but tender texture, as a side effect, their nutritional and health outcomes may also be significantly affected. First of all, as a consequence of dehydration, nutrient density notably increases, so that meat products processed that way have a higher content of some nutrients in which meat is rich, such as proteins, iron, zinc, niacin, pyridoxine or cobalamin. Nevertheless, other compounds, e.g., ubiquinone (coenzyme Q10) with health properties tend to decrease or even disappear during the ripening process [49].

Secondly, the extensive proteolysis during the maturation, as a result of endogenous and microbial proteases, leads to high levels of free amino acids and peptides with large differences in molecular weight [50]. In turn, this leads to faster amino acid uptake rates during digestion (additional compared to regular cooking), which has been linked in some cases to higher anabolic potential for protein-rich foods [51]. On top of that, some of these new generated peptides show bioactive properties, mainly antihypertensive and antioxidative in hypertensive rats [52]. Human studies have also demonstrated prolonged gastric emptying and increased satiety [53]. It is well known that during meat protein digestion, peptides with bioactive properties are released. In the case of aged meat products, these proteolytic processes already take place during the ripening, and as a consequence, such peptides are already present in the product before human digestion. The extent of proteolysis, the type of enzymes involved and the raw material strongly influence the type, number and quantity of bioactive peptides generated in these ripened meat products. Thus, it has been shown that 24-month ripened Iberian ham contains higher levels of highly active angiotensin-converting enzyme inhibitory activity than dry-cured hams processed for shorter times [54]. Such bioactive peptides have also been identified in aged beef [55], aged duck [56] and dry-fermented sausages [57]. In fermented products, it has been evidenced that the type of starter culture is related to the type, the amount and activity of these bioactive peptides [58]. It has been hypothesized that the presence of antihypertensive peptides might counteract the effect of their high salt content on blood pressure; however, studies to document their effects in humans are still missing.

The consumption of hydrolyzed proteins has been linked to other potential positive health outcomes, such as the regulation of bile acid metabolism [59] and induced satiety [60]. In fact, meat hydrolysates have been shown to increase the release of cholecystokinin [61], a gut peptide hormone inducing satiety: this may lead to smaller and less frequent meals and eventually to a lower dietary intake.

Lactic acid bacteria are commonly used as starter cultures for the production of fermented meat products due to their distinct biochemical effects, mainly lactic acid generation, pH drop, flavour generation and bio-protective effects [62]. In fact, the traditional production of dry-fermented products was based on the fermentation of added sugars by naturally present lactic acid bacteria. Some of the commercial starter strains and also some indigenous isolates from dry sausages have shown probiotic properties. In fact, since these products are not heat-treated, they provide suitable conditions required for the survival of probiotics. Additionally, it seems that the meat product matrix may help probiotics to survive through the gastrointestinal tract [63]. Moreover, there have been numerous attempts to select and use probiotic bacteria adapted to the harsh conditions of dry-fermented sausages (high salt, low aw, low pH, low sugar content, nitrites, etc.). Naturally occurring bacteria in sausages are mostly strains of lactic acid bacteria with a high degree of hydrophobicity, which usually is linked to probiotic potential. For example, strains of *Lactobacillus sakei*, *L. curvatus*, *L. plantarum*, *L. brevis*, *L. fermentum*, *L. lactis*, *L. pentosus*, *Pediococcus acidilactici* or *P. pentosaceus*, isolated from Scandinavian, Greek, Spanish or other commercial fermented sausages, have been characterized as probiotic [64]. Other types of added probiotic bacteria have difficulties in surviving in the dry-sausage environment.

On the negative side, the high salt content and the presence of nitrites in this type of meat products have been pointed out as potential causative factors in developing hypertension and colorectal cancer, respectively. It remains to be investigated whether the presence of antihypertensive peptides may counteract their effect on blood pressure in humans. On top of that, the amount of salt in processed meat products has steadily decreased in the UK during the last few decades [65]. Going further in this direction appears potentially problematic, since lower levels may imply microbiological risks and texture defects, and salt substitutes, e.g., calcium and potassium salts, tend to confer an unpleasant taste [65]. As far as nitrites are concerned, their role in cured products is crucial in controlling microbial growth (especially that of *Clostridium botulinum*), stabilizing color and promoting the formation of a characteristic flavor [66]. On the other hand, their presence in foods may lead to the formation of carcinogenic N-nitrosamines. While this has been experimentally proven, the levels of such compounds are quite low or even non-detectable in non-heated products, such as dry-cured and dry-fermented sausages. In addition, the common use of high amounts of ascorbic acid in these products strongly limits the formation of these harmful compounds [65].

## 5. Fortification of Meat Products

An approach that has been taken to combat potentially harmful effects associated with the ingestion of processed meat is to fortify processed meat products with ingredients that may counteract or neutralize such negative health effects. There is extensive evidence that intake of dietary fibers is associated with beneficial effects on gut health. Using a rat model, it was recently shown that fortification of pork sausages with inulin resulted in significant effects on the metabolites generated in the gastrointestinal tract by the gut microbiome [67]. Thus, fortification of processed meat with inulin enhanced the formation of acetate, propionate and butyrate, the characteristic short-chain fatty acids that have been identified as pivotal in the beneficial effects associated with dietary fiber consumption [68,69]. In a human intervention study, Perez-Burillo and colleagues [70] also showed that inclusion of dietary fiber in a fermented meat product (salami) stimulated the formation of butyrate upon ingestion. Furthermore, it has also been shown that including butyrylated starch in the diet enhances short-chain fatty acid content in the gut and attenuates the formation of unwanted O6-methyl-2-deoxyguanosine adducts, which is known as toxic and mutagenic modification, and found to be associated with high red meat intake [71]. Consequently, current knowledge indicates that fermentable dietary fibers and short-chain fatty acid-containing compounds can counteract the potential harmful effects in the colon associated with intake of processed meat. Unfermentable dietary fiber is less explored, but in animal model studies, they also seem to have considerable potential in cancer prevention [72].

Intriguingly, cohort studies also point at a high calcium intake having a positive effect on colon health [73,74]. Using a rat model, Thøgersen and colleagues [67] recently investigated the effect of fortifying processed meat with calcium and inulin in combination or alone. Interestingly, addition of calcium-rich milk minerals significantly reduced both the formation of unwanted N-nitroso compounds in the gastrointestinal tract when compared with ingestion of non-fortified processed meat and stimulated the formation of short-chain fatty acids in the colon [67]. Consequently, promising results reveal that potential harmful effects associated with meat ingestion in fact can be mitigated through modulation of the meat product matrix and fortification of meat products or strategic design of meals with the inclusion of components such as dietary fiber and calcium that neutralize unintended effects in the gastrointestinal tract associated with meat intake.

## 6. What Do We Know and Not Know on the Food Matrix

The food matrix can be defined as the nutrient and non-nutrient components of foods and their molecular relationships, i.e., chemical bonds, to each other [75]. Nutrients are seldom present in a free form, but are incorporated into larger molecules or embedded in granules or specific compartments. This association with other constituents of the food affects the release of the nutrients from the food and thereby both the accessibility and bioavailability of any given nutrient [76,77]. In other words, it is not the total amount of a nutrient ingested that determines the amount absorbed, but the food matrix, interaction between nutrients and host related factors. The food matrix directly affects the digestion and absorption of the nutrients in the gastrointestinal tract.

In the past, the nutritional quality of a food was associated with the total amount of nutrients; however, due to food matrix effects, the amount absorbed actually differs between foods despite having equal contents. Several examples of food matrix effects are known for plant foods; the best-known examples are probably the phytate–mineral interactions, where minerals are tightly bound to phytate and only released upon degradation (fermentation or soaking) of the phytate, and carotenoids, which are released from plant cells by cutting or chopping the vegetables [78], by being solubilized into lipids in the food matrix and by several other factors [79]. Another intriguing example is the absorption of carcinogens, including food mutagens from fried meat, onto chlorophyll; this absorption has been shown for aflatoxin B1 to be sufficiently strong to reduce DNA damage in humans [80,81]. This observation also further underlines the importance of ingesting highly proteinaceous foods together with a complex food matrix including fresh greens. In relation to meat, cooking reduces the amount of fat, peptides and vitamins while increasing the concentration of some minerals, e.g., Zn and Fe (particular in beef), while the effect on Ca and Mg is inconclusive [82,83]. In addition to heme-iron being better absorbed than non-heme-iron, and red meat therefore being a superior source of iron [28], ingestion of supplemental prebiotics increases the absorption of heme-iron from beef [84], suggesting that, e.g., inulin fortification or fermentation of meats may further increase iron availability and potentially that of other minerals. In all cases, preparation of the food by heating, chopping or fermentation may liberate or release the nutrients and non-nutritive compounds from the food matrix and thereby improve or reduce their bio-accessibility, depending on the meal composition.

Food matrix effects are important, but meal composition, as well as interactions between foods in the meal, also affect bio-accessibility and bioavailability. The ‘meat factor’, whatever it is, is an example [29]. When consuming meals composed of both vegetables and meat, the meat factor promotes the absorption of non-heme iron from the plant products [30].

## 7. Meat and Chronic Disease—How Good Is the Evidence?

Due to limitations in the duration of intervention studies needed to measure chronic disease endpoints, most studies on the effects of meat consumption on health outcomes, such as cardiovascular disease (CVD) and cancer, are observational. The number of studies is high and systematic reviews and meta-analyses have therefore been conducted repeatedly by different groups. However, conclusions are divided and the issue therefore controversial.

### 7.1. Meat and Cancer

In the continuous update project [85] on colorectal cancer risks, the evidence for an effect of red as well as processed meat intake has been judged as strong, but the overall conclusion was graded in that the evidence for processed meat was classified as *sufficient*, while that for red meat was classified as *probable*. This was based on overall limited heterogeneity of the studies included in the analysis, no observed small-study bias, significant dose–response and plausible mechanisms. The grading of the evidence for red meat was decreased from *sufficient* in 2007 to *probable* in 2018. This may have been caused by published meta-analyses failing to show a significant overall effect and geographical differences with significant effects observed in Europe but not in the Americas or Asia. These conclusions are corroborated by similar findings in several recent meta-analyses [12,86,87]. However, some meta-analyses report similar magnitudes and trends but conclude that the magnitude of the cancer-causing effect is limited and the evidence as weak and likely to be affected by significant heterogeneity and confounders [15,16]. Uncertainty as to the classifications of meat into red and processed meat, interactions with other dietary factors and geographical variations are some of the factors described as potential confounders. While official recommendations in most countries support reductions in red and processed meat intake based on the findings by international organizations, there is obviously some scientific controversy as to the technical judgement of the quality of evidence and the impact of decreased intakes on colorectal cancer risk. Some of this could be resolved by better biomarkers of red and processed meat intake [88,89] as well as biomarkers related to their potential mechanisms of action, which should help in removing potential confounding factors.

### 7.2. Meat, Cardiovascular and Chronic Disease

Händel and colleagues performed an umbrella review of systematic reviews on associations between processed meat intake and morbidity and mortality of chronic diseases [14]. The quality of the systematic reviews reporting positive associations between processed meat intake and the risk of various cancers and cancer mortality, type 2 diabetes and CVD, and CVD mortality was moderate, and the overall certainty in the evidence was very low across all individual outcomes, due to a serious risk of bias and imprecision. The results of the generally more biased case–control studies were more likely to suggest a positive association than the results from cohort studies.

In a systematic review and linear dose–response meta-analysis of prospective studies, Schwingshackl and colleagues found a positive association between hypertension and intake of red meat (relative risk 1.14 per 100 g/day; 95% confidence interval (CI): 1.02, 1.28) and of processed meat (relative risk 1.12 per 50 g/day; 95% CI: 1.00, 1.26) [90]. However, the authors conclude that the overall quality of the meta-evidence for the association in the studies included was of low quality.

Lippi and colleagues found no clear association between red meat consumption and ischemic heart disease in a systematic review of prospective cohort and case–control studies due to the large heterogeneity of the criteria used for defining red meat and diagnosing ischaemic heart disease [91].

A recent systematic review and meta-analysis on associations between red and processed meat intake and risk of heart failure found no association for highest versus lowest red meat intake (relative risk 1.04; 95% CI: 0.96–1.12), but a positive association for processed meat intake (relative risk 1.23 per 50 g/day; 95% CI: 1.07–1.41) [92]. Unfortunately, the quality of the included studies was not graded. Subgroup analyses showed a significant association between processed meat intake and heart failure among Europeans (relative risk 1.33 per 50 g/day, 95% CI = 1.15–1.54), but not among Americans. No association was found between heart failure risk and red meat intake in either continent [92].

Neuenschwander and colleagues found a positive association in dose–response studies of processed red meat (hazard ratio 1.44; 95% CI: 1.18–1.76), processed meat (hazard ratio 1.37; 95% CI: 1.22–1.54), and bacon (hazard ratio 2.07; 95% CI: 1.40–3.05) intake and risk of type 2 diabetes in an umbrella review of prospective cohort studies [93]. No significant association was found for unprocessed red meat (hazard ratio 1.11; 95% CI: 0.97–1.28). The methodological quality of the meta-analyses was mostly high, but the quality of evidence was low for unprocessed red meat, moderate for processed red meat and high only for processed meat and bacon.

### 7.3. Interpretation of Observational Studies

When assessing results in meta-analyses, the data are only as valid as each individual study. Differences in the definition of which products to include in the categories of meat and processed meat (and exclusion of specific meat products [94]), and differences in serving sizes among countries play an important part in the validity and interpretation of the results. Equally important are the characteristics, medical history and total dietary intake of the participants included in the studies; factors influencing the results but, despite several statistical models, close to impossible to eliminate.

Overall, the observational evidence for the effects of red meat on chronic disease is weak and methodological issues have downgraded the overall judgment, although the direction of the effect for colorectal cancer is quite consistent. The evidence for adverse effects of the heterogeneous group of processed meat is moderate-to-strong for several endpoints with colorectal cancer as the most important effect. Scientific disputes exist regarding the consistency of the evidence for most endpoints. Better insights and tools such as biomarkers to support accurate intake assessments [88,95,96], discrimination between different groups of processed meats and assessment of mechanisms in cancer development are likely to resolve some of this controversy. The potential nutritional and mechanistic confounders are discussed in the following section.

## 8. The Importance of Confounders and Co-Factors

When estimating associations between meat intake and disease risk by comparing groups with high and low meat intake, respectively, it is pivotal to be aware which foods substitute meat in the low-meat diet. High meat intake is not necessarily confounded by an unhealthy diet, e.g., low in fruit, vegetables, whole-grain and dietary fiber intake and high in sugar and alcohol [97]. However, it was observed in analyses of dietary patterns in adult Danes that the 25% of the population with the highest reported meat intake along with an unhealthy diet (the highest quartile) have a red meat intake that is significantly higher (approximately 20% higher) than the 25% of the population with highest meat content in combination with a healthy diet (144 g/10 MJ compared with 121 g/10 MJ) [98]. For processed meat, the difference is even higher (32%; 87 g/10 MJ for those with an unhealthy diet compared with 66 g/10 MJ along with the healthy diet). This was also observed in an Irish study where a high intake of processed meat was associated with a low intake of fruit, vegetables, fish and whole grain, indicating a less healthy diet [94]. Thus, comparing disease risk in groups with high and low meat intake without corrections for dietary quality will inevitably be a comparison of unhealthy and healthy diets if no or inappropriate corrections for dietary quality are made. Moreover, the groups with high meat intake along with an unhealthy diet were shown to have a significantly higher dietary intake of foods which may have the potential to increase disease risk (e.g., fried potatoes, high-fat gravy, fatty spreads and fast foods) when compared with groups with high meat intakes as part of a healthy diet [98].

Many cohort studies present estimates including both a basic model with corrections for only basic confounders, e.g., age, sex and energy intake, and a more extended correction, e.g., body mass index, smoking habits, social status, and intake of healthy foods such as fruit, vegetables and whole grains. However, it can be questioned whether such correction are sufficient to take into account all differences in dietary quality that accompany high and low dietary meat content. In addition, it can be questioned whether corrections for too many confounders will interfere with the actual effects examined. However, it is not unusual that after extensive corrections for confounders, the associations found in the more basic model are no longer present [99], indicating that the corrections strongly modulate the estimates.

## 9. Research Gaps and Recommendations

A summary of recommendations and identified issues relevant for future research is presented in Table 1.

**Figure 2:**
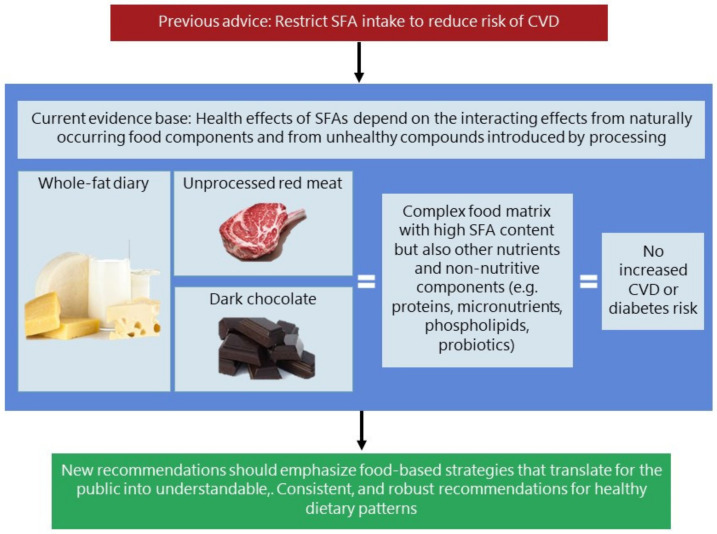
Shifting from saturated fatty acid-based to food-based dietary guidelines for cardiovascular health. CVD, cardiovascular disease; SFA, saturated fatty acid. Used with permission from Astrup et al. 2020 [10].

Emerging evidence indicates that foods cannot just be viewed as sources of specific nutrients, rather as a totality of several nutrients and other components that exert an effect depending on the composition, processing, meal composition and consumer habits (Figure 2). As an example, the effect of SFA from butter differs from that of similar SFA in fermented dairy products [9,10,100]. This is an effect which, to an extent, may be explained by different low density lipoprotein (LDL) particle sizes being affected differently by SFA intake [101,102] or by the differences in content of dairy calcium. Analysis of the total number of LDL particles is commonly used to evaluate CVD risk, but particularly small LDL particles seems to be highly correlated with CVD whereas the larger LDL particles are not. Future studies should include analyses and a presentation of the different LDL particle sizes in order to separate the specific effect. In addition to the effect of SFA intake, the pathophysiological effects of salt and other additives from industrial processing are yet to be identified [103].

When viewing the baseline characteristics of participants in two large cohorts according to quintiles of total red meat consumption, it becomes clear that those with the highest meat consumption also have a lower consumption of fish, vegetables and whole grains [4,17], pointing towards a lower intake of several kinds of dietary fiber among these meat-eaters. Other studies also found those with a higher intake of meat to have a less healthy eating pattern [98], suggesting that an effect may be due to the absence of dietary fiber or other plant components more than the intake of meat per se, exerting an effect of health parameters. The positive effect of dietary fiber on human health is well established; for example, a change to a more healthy diet is shown to improve the gut microbiome and functionality independently from energy intake [25]. However, studies with equal meat contents are lacking. A high-quality human intervention study investigating the effect of processed meat with and without appropriate types of dietary fiber in humans could elucidate the effect on risk markers of CVD and microbiota and evaluate whether the absence of dietary fiber negatively influences the metabolic effects after the consumption of processed meat.

Despite the large body of observational studies on meat consumption and health outcomes, confounding factors and different or undefined subgrouping of meat types make it difficult to evaluate to what extent residual confounders might explain the modest increases in risk observed in association with red and processed meat intake. We therefore advocate for the completion of randomized controlled interventions of high quality to assess the effect of pre-defined meat consumption on relevant validated biomarkers among healthy people as well as among those at risk of CVD, type 2 diabetes and cancer (especially colorectal cancer).

In conclusion, meat is a source of high-quality proteins, minerals and vitamins and other compounds, difficult to obtain in sufficient amount from other sources. The current available research is inconclusive and does not support that meat consumption as part of a healthy diet increases the risk of disease. Moreover, considering the potential confounding factors and lack of interventional studies, there is a need for sufficiently powered randomized controlled trials assessing the effect of meat consumption on shorter-term risk markers. While several biomarkers exist and have been partially validated according to a currently proposed standard [104], additional work is needed for their full validation [88,89,95,96]. Good biomarkers to assess intakes of different meats and of potentially protective dietary components in observational studies is another need to resolve the effect and confounders. In addition, mechanistic studies to therefore identify pathways and identify potential fermentation and processing methods increasing nutrient availability and effect are warranted.

## Figures and Tables

**Table 1:** Summary of recommendations and future research.

*Recommendations* Standardization of the definition of red, processed and unprocessed meat products Completion of randomized controlled studies with a solid methodological approach to thoroughly examine and identify the pathophysiological effects of: Different types of fresh meats; red and white Fermented meat products (dry cured meats) Other processed meat products To investigate the metabolic effects of consuming meat as part of a healthy diet Improve the identification of metabolic changes in response to meat consumption, including biomarkers of intake and effect. *Future strategies* Future studies should identify a possible threshold for apparent healthy factors that become unhealthy when consumption increases beyond a certain level—can this level be influenced by intake of other foods/nutrients, e.g., does a high intake of dietary fiber make you more robust and resilient to a high intake of meat? Do processed meat products fortified with, e.g., dietary fiber or calcium exert an effect different from regular processed meat? Does fresh minced meat exert an effect different from regular fresh meat? Assess the effect of different amounts of meat consumption as part of a healthy diet in a healthy population as well as in those with overweight and obesity and thereby at risk of CVD and type 2 diabetes Characterization of nutrients and non-nutritive compounds in processed meat, wet and dry cured How does processing/fermentation affect content and bioavailability of nutrients? Including partly liberation of nutrients from connective tissues. Link to/investigation of expected biological effects Include identification of different lipoprotein particle sizes when analyzing changes in plasma cholesterol
